# Case Report: Integrating clinical presentation and genetic analysis in P450 oxidoreductase deficiency: a novel mutation and systematic review

**DOI:** 10.3389/fendo.2026.1791297

**Published:** 2026-04-10

**Authors:** Chen Zhang, Zhanbo Zhao, Yi Du, Qi Huang, Yushu Liu, Gubing Fu, Jianglong Xu

**Affiliations:** Department of Orthopedics, Shenzhen Children’s Hospital, Shenzhen, China

**Keywords:** cytochrome P450 oxidoreductase deficiency (PORD), endocrinology, infant, novel mutation, *POR* gene, skeletal abnormalities

## Abstract

**Background:**

Cytochrome P450 oxidoreductase deficiency (PORD) is an ultra-rare autosomal recessive disorder caused by mutations in the *POR* gene and characterized by highly heterogeneous skeletal, genital, and endocrine manifestations. Owing to this complexity, PORD remains frequently underrecognized in clinical practice, and integrated clinical–genetic syntheses remain limited.

**Methods:**

A retrospective analysis was conducted on the clinical data of a PORD patient treated at Shenzhen Children’s Hospital. Relevant literature was retrieved from PubMed, Web of Science, and China National Knowledge Infrastructure (CNKI). Reported cases were analyzed with respect to sex, age, geographic distribution, clinical manifestations, and *POR* gene variants.

**Results:**

The patient from our hospital, a 7-month-old infant, presented with characteristic features including frontal bossing, craniosynostosis, flat nasal bridge, proximal radioulnar synostosis, clitoromegaly, partial labial fusion, and steroid hormone abnormalities. Genetic testing identified compound heterozygous variants, p.G146fs*111, a novel mutation, and p.R457H. The patient underwent bilateral mandibular distraction osteogenesis and cranial reconstruction, which alleviated airway obstruction, swallowing difficulty, and craniosynostosis. A total of 50 eligible studies were identified, comprising 167 patients (male:female = 77:90). The major clinical findings were skeletal deformities in 124 cases (74.25%), gonadal deformities in 121 (72.46%), hormonal abnormalities or delayed puberty in 127 (76.05%), and adrenal insufficiency or crisis in 108 (64.67%). Additionally, ovarian cysts were observed in 36 female patients (40.00%). Among all patients, the allele frequency of the p.R457H variant was 27.25%, while that of the p.A287P variant was 14.97%. In the 22 Chinese patients, the allele frequency of the p.R457H variant reached 38.64%.

**Conclusion:**

We report the clinical features of a PORD patient carrying a novel *POR* mutation, p.G146fs*111. PORD typically presents with skeletal and genital malformations as well as adrenal insufficiency. Management requires multidisciplinary collaboration, including individualized steroid replacement, regular blood pressure monitoring, and surgical intervention when necessary. The p.R457H variant may represent a hotspot mutation in East Asian populations.

## Introduction

**Figure 1:**
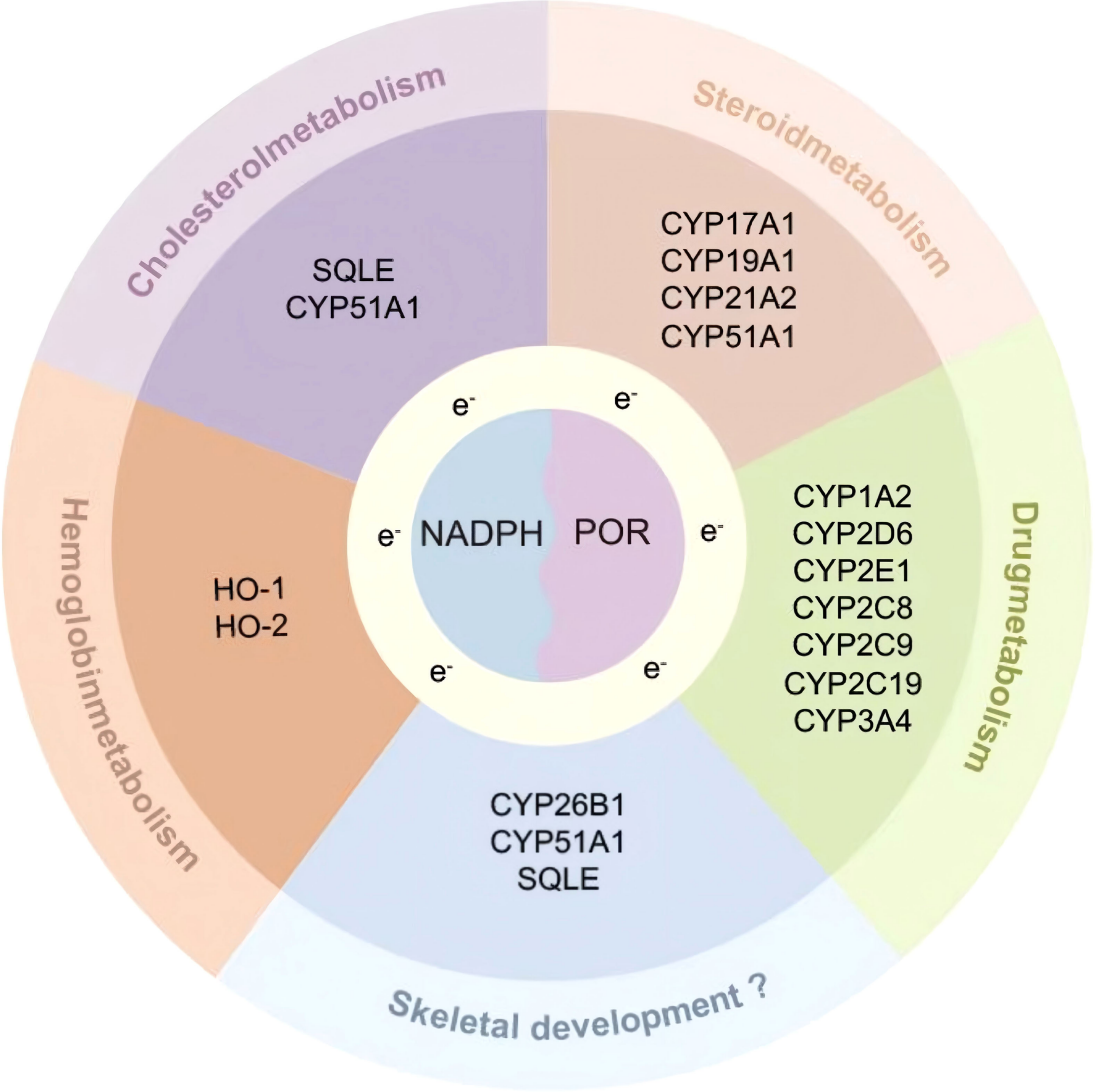
Schematic diagram of POR involvement in multiple metabolic pathways.

Cytochrome P450 oxidoreductase deficiency (PORD) is an ultra-rare autosomal recessive disorder. Since its initial identification in 2004, over 100 cases have been documented globally (1–3). The cytochrome P450 oxidoreductase (POR) protein, comprising 680 amino acids, functions as an essential electron donor. It transfers electrons from nicotinamide adenine dinucleotide phosphate (NADPH) to more than 50 microsomal cytochrome P450 (CYP) enzymes (4–6). These enzymes are critical for steroid and cholesterol biosynthesis, drug metabolism, and various other physiological processes (**Figure 1**). Mutations in *POR* reduce or abolish the activity of its dependent enzymes, leading to a broad spectrum of clinical manifestations. These include genital abnormalities, steroid hormone dysregulation, infertility in both sexes, and skeletal malformations resembling Antley-Bixler syndrome. Notably, the clinical phenotype exhibits significant variability depending on the specific *POR* mutations, contributing to frequent misdiagnosis or underdiagnosis in clinical practice (7). To date, systematic reviews of PORD cases remain limited, often outdated, and typically include only small patient cohorts, thus offering limited guidance for clinical decision-making.

Here, we report a 7-month-old female patient diagnosed with PORD, presenting at birth with multiple skeletal malformations and genital anomalies. She required two surgical interventions to address respiratory compromise, feeding difficulties, and craniosynostosis. Whole-exome sequencing (WES) of the proband and her parents revealed previously unreported compound heterozygous mutations in the *POR* gene. Furthermore, we conducted a comprehensive, systematic review and analysis of the existing PORD literature. This integrated approach aims to enhance the understanding of PORD’s phenotypic spectrum and genetic basis, providing valuable insights for future diagnostic and therapeutic decision-making.

## Methods

### Clinical data collection

Written informed consent was obtained from the patient’s legal guardian for the publication of this case report, including all clinical details and accompanying images. The collected clinical data encompassed: Demographic characteristics, Comprehensive physical assessment findings, Imaging studies, Laboratory analyses, Therapeutic interventions and treatment response, Historical medical records, Parental health status and maternal medication history during gestation, Prenatal screening/diagnostic records, and Longitudinal follow-up data.

### Whole-exome sequencing

Genomic DNA was extracted from peripheral venous blood samples of the proband and her parents using the QIAamp DNA Mini Kit (Qiagen, Shanghai, China). DNA concentration was quantified with a Qubit 3.0 Fluorometer (Thermo Fisher Scientific; Cat. No. Q33216) and the Qubit dsDNA BR Assay Kit (Cat. No. Q32850). DNA integrity was verified by 1% agarose gel electrophoresis. Qualified DNA was fragmented to an average size of 150 bp using a Covaris S220 Focused-ultrasonicator (Covaris, MA, USA). Libraries were prepared with the SureSelectXT Reagent Kit (Agilent; Cat. No. G9611B), Agilent V6 Exome Probes (Cat. No. 5190-8863), and Agencourt AMPure XP Beads (Beckman Coulter; Cat. No. A63881) following manufacturers’ protocols. Paired-end sequencing (150-bp reads) was performed on an MGISEQ-200RS platform (MGI Tech Co., Ltd.) using the MGISEQ-200RS High Throughput Sequencing Kit (Cat. No. 1000012555). Sequencing quality control metrics included: Average target region coverage depth ≥150×; 95% of target bases covered at >20× depth.

### Analysis and evaluation of variants

Whole-exome sequencing data analysis was performed by first processing raw sequencing data through adapter trimming and low-quality read filtration using Trimmomatic (v0.39), followed by alignment of the filtered reads to the UCSC hg19 reference genome with Burrows-Wheeler Aligner (BWA-MEM v0.7.17). Variant calling was conducted using the Genome Analysis Toolkit (GATK v4.2.6.1) pipeline, which included base quality score recalibration (BQSR), single-nucleotide variant (SNV) and insertion/deletion (INDEL) calling via HaplotypeCaller, and joint genotyping across trios using GenomicsDBImport and GenotypeGVCFs. Exon-level copy number variation (CNV) detection was carried out with ExomeDepth (v1.1.16), and variant annotation was performed using ANNOVAR (2020) integrated with RefGene, ClinVar, and dbNSFP v4.3a databases. For variant prioritization, we first excluded variants with minor allele frequency (MAF) ≥0.005 in any population database (1000 Genomes Phase 3, ESP6500, ExAC v0.3.1, gnomAD v2.0.1, or gnomAD East Asian [gnomAD-EAS]). Subsequently, functional predictions were applied: missense variants were evaluated using SIFT (v6.2.1), PolyPhen-2 (v2.2.2), MutationTaster (2021; disease-causing), and CADD (v1.6); splicing variants were assessed with SpliceAI (v1.3.1) and dbscSNV (ADA/RF); and evolutionary conservation was analyzed using PhyloP (hg19.100way) and GERP++ (hg19). Finally, pathogenicity assessment integrated all evidence tiers following ACMG/AMP 2015 guidelines.

### Sanger sequencing

Sanger sequencing was employed to validate the candidate variants identified through whole-exome sequencing. PCR primers flanking each target variant were designed using Primer3 web software (v.0.4.0; http://primer3.ut.ee/). The resulting PCR products were purified and subsequently sequenced in both directions using the BigDye™ Terminator v3.1 Cycle Sequencing Kit (Applied Biosystems; Thermo Fisher Scientific, Inc.) on an ABI 3130xl Genetic Analyzer. Sequence chromatograms were analyzed and aligned to the reference sequence using Sequencher software (v5.4.6; Gene Codes Corporation).

### Systematic review

**Figure 2:**
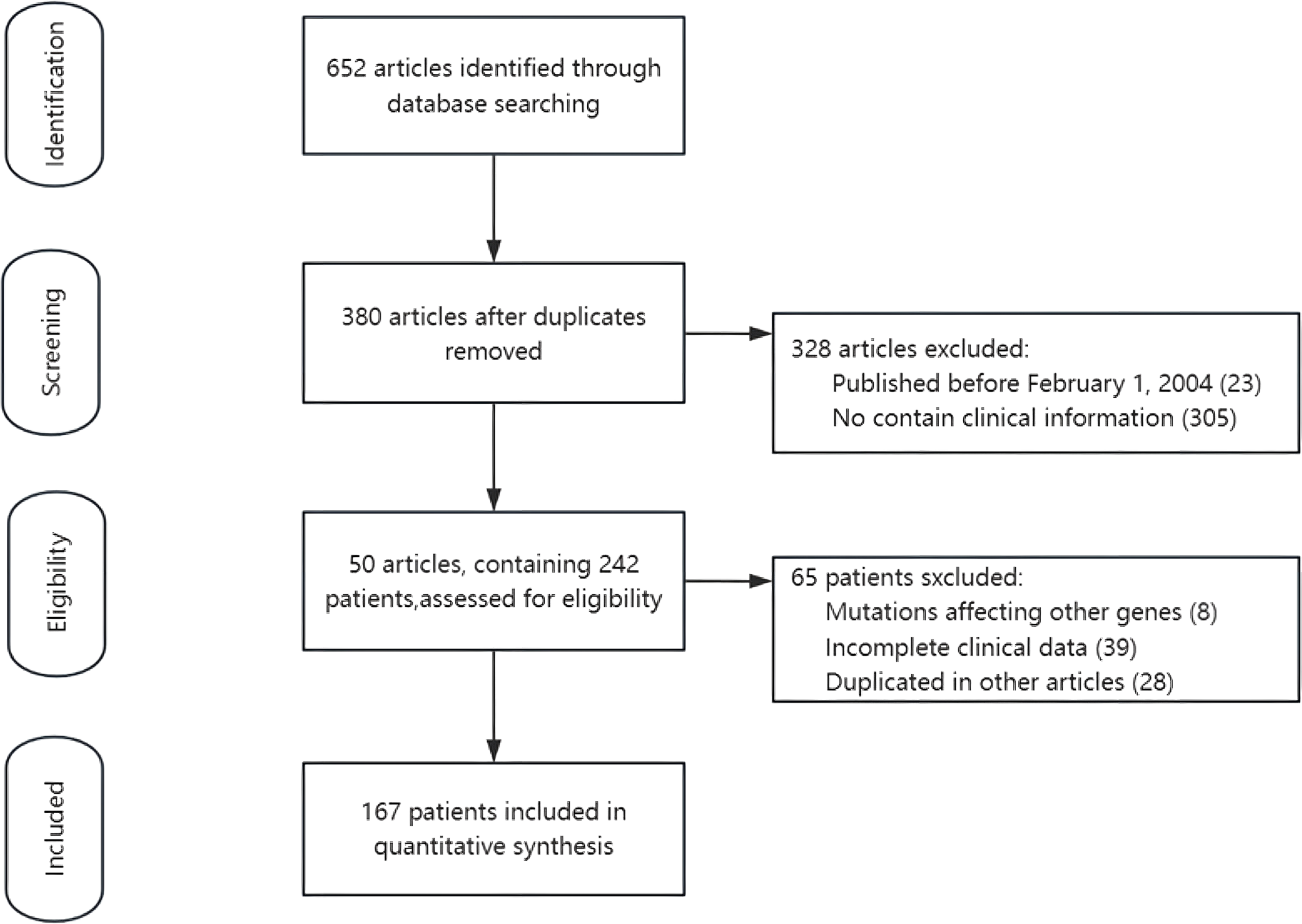
Flowchart of the included studies.

We conducted a systematic literature review by searching the PubMed, Web of Science, and China National Knowledge Infrastructure (CNKI) databases to identify all published case reports and case series related to P450 oxidoreductase deficiency (PORD). The search encompassed all records from the inception of each database through May 1, 2025. The search strategy utilized the following key terms: (“P450 oxidoreductase” OR “POR gene”) AND (“deficiency” OR “deficient” OR “mutation” OR “congenital adrenal hyperplasia” OR “DSD” OR “pregnancy” OR “heterozygosity”). After removing duplicate records, studies were excluded based on the following criteria: (i) publication prior to February 1, 2004, corresponding to the period before the formal identification of PORD; (ii) lack of detailed clinical patient information. During patient selection, we further excluded individuals with: (i) incomplete clinical data; (ii) confirmed pathogenic variants in other genes associated with overlapping dysmorphic features; or (iii) duplicate reporting in multiple publications. Following this screening process, a total of 167 unique PORD patients with sufficient clinical information were included from the eligible publications (**Figure 2**). Study selection and data extraction were independently performed by two authors, with discrepancies resolved by consensus.

## Results

### Clinical findings

A 7-month-old infant was referred to our hospital for evaluation of craniofacial abnormalities. The patient was delivered via Cesarean section at 38 weeks and 4 days of gestation due to oligohydramnios. Apgar scores were 10 at both 1 and 5 minutes. Physical examination revealed dysmorphic craniofacial features, including midface hypoplasia, frontal bossing, hypertelorism, and a depressed nasal bridge. Due to severe respiratory and feeding difficulties, the patient underwent bilateral mandibular distraction osteogenesis at a local hospital at age 1 month and 22 days. The distraction devices were subsequently removed at 6 months and 16 days of age, resulting in marked improvement in both respiratory and feeding function. The mother was a 35-year-old primigravida with no history of teratogenic medication exposure or radiation during pregnancy. Notably, she developed signs of virilization during the second trimester, including hirsutism and acne, which resolved gradually after delivery. No family history of genetic or metabolic disorders was reported.

### Physical examination

**Figure 3:**
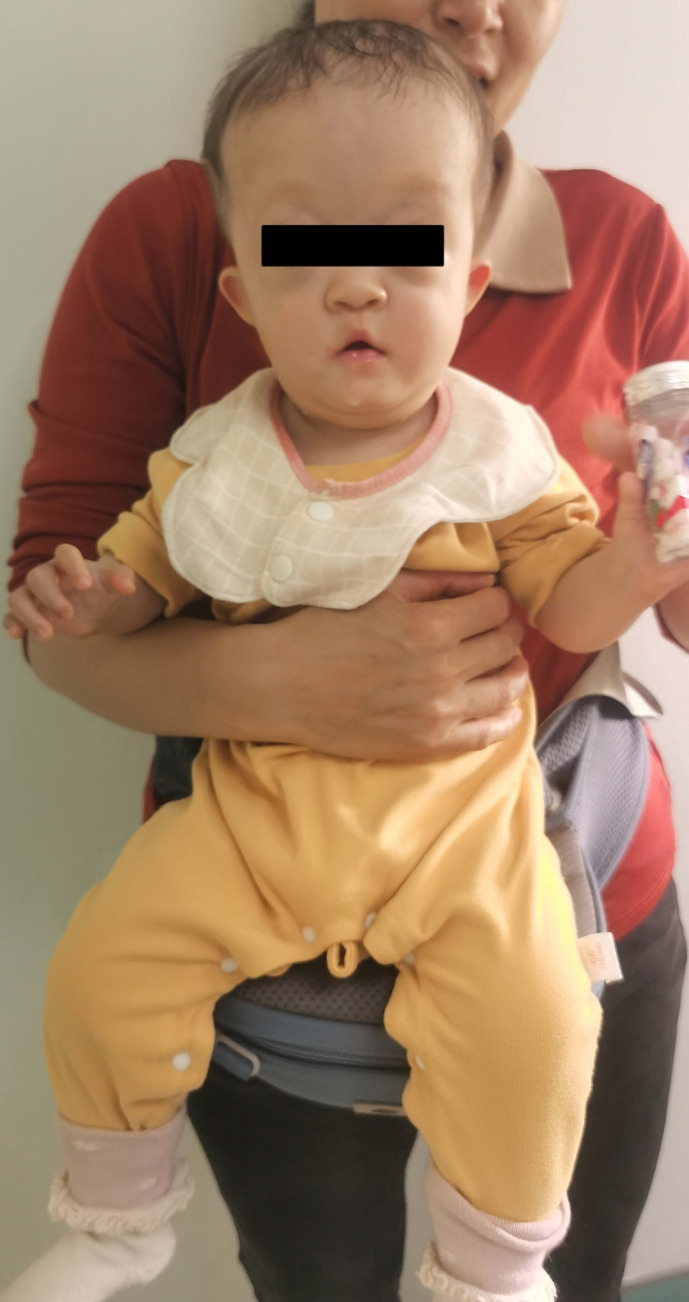
Full-body appearance of the patient.

Physical examination indicated a body length of 67.0 cm and weight of 6600 g, with fair nutritional status. The anterior fontanelle showed mild fullness, and the left coronal suture was synostosed, resulting in brachycephaly and frontal bossing. Craniofacial dysmorphism included bilateral proptosis, hypertelorism, a depressed nasal bridge, and a pear-shaped nose. The ears were low-set and posteriorly rotated, with underdeveloped antihelices and a single horizontal earlobe crease. Audiological assessment revealed moderate hearing impairment in the left ear and mild impairment in the right. Orthopedic evaluation showed bilateral elbow contractures fixed at 90°flexion, with complete loss of extension and flexion. The humeroradial joint could not be palpated, and bony fusion between the proximal radius and the humerus was noted. The forearms were held in neutral position with approximately 30° pronation and absence of supination. Wrist examination was unremarkable. Both thumbs were absent of nails, though all other digits were normal in morphology and function. Similarly, the great toenails were absent bilaterally, with normal appearance and mobility of the remaining toes. The spine and other limb segments showed no deformities. Genital examination revealed mild clitoromegaly with partial labial fusion. Examinations of the neck, chest, abdomen, and nervous system were within normal limits (**Figure 3**).

### Laboratory and auxiliary examination

The initial hormonal profile was notable for several abnormalities: serum cortisol 0.5 μg/dL (normal range: 0.5-16.6 μg/dL), testosterone 0.02 ng/mL (normal: 0.03–0.48 ng/mL), adrenocorticotropic hormone (ACTH) 92.56 pg/mL (normal: 7.2–63.3 pg/mL). Progesterone 9.6 ng/mL (normal: 0.20-1.50 ng/mL) and 17-hydroxyprogesterone (17-OHP) 4.3 ng/mL (normal: 0.29-1.8 ng/mL). In contrast, aldosterone levels, thyroid function tests, and neonatal screening for inborn errors of metabolism were all within normal ranges. Preoperative investigations, including assessments of liver and renal function, serum electrolytes, and coagulation profiles, revealed no abnormalities.

**Figure 4:**
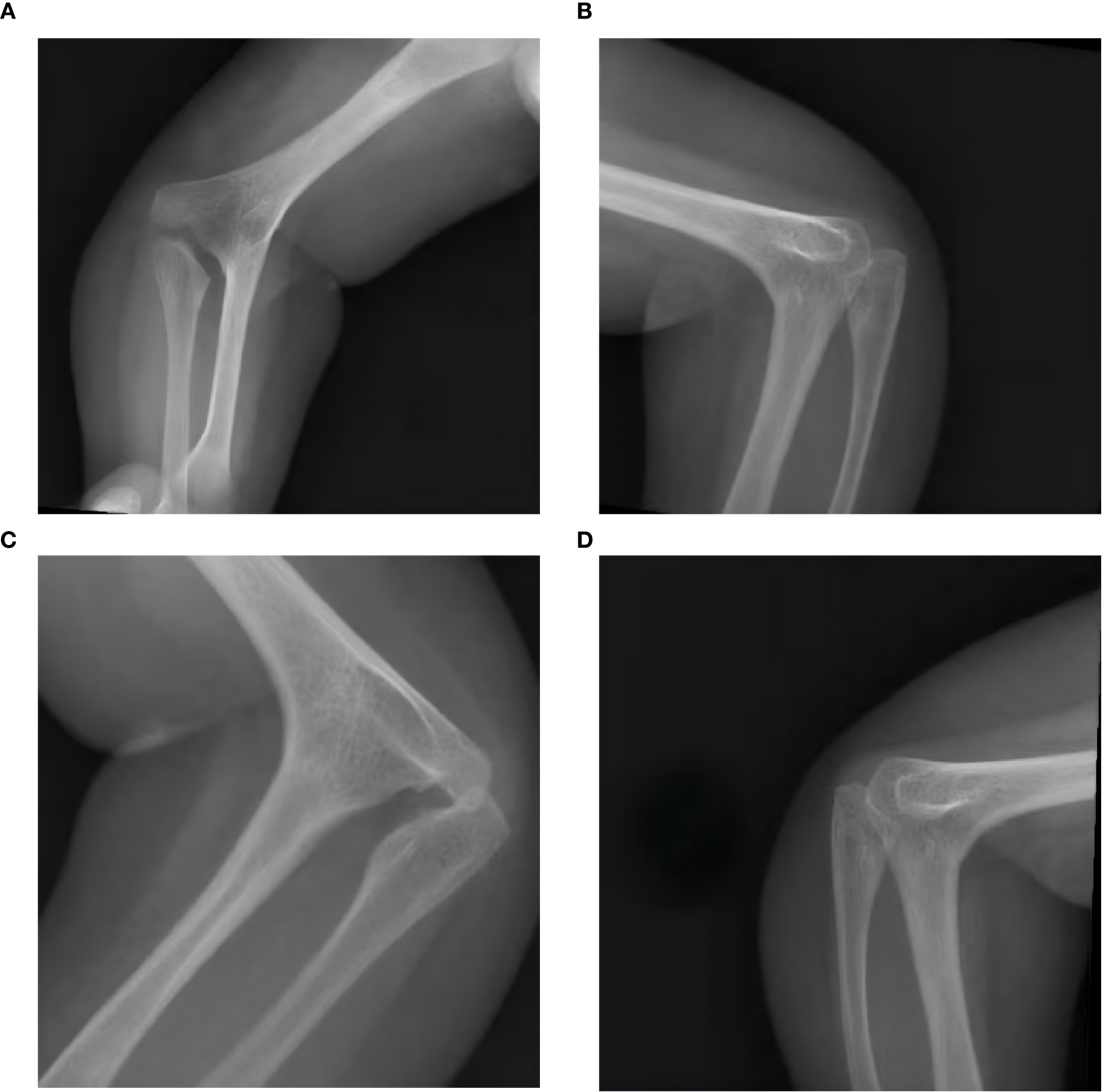
Bilateral elbow joint X-ray of the patient [**(A)** Anteroposterior view of the left elbow; **(B)** lateral view of the left elbow; **(C)** anteroposterior view of the right elbow; **(D)** lateral view of the right elbow].

**Figure 5:**
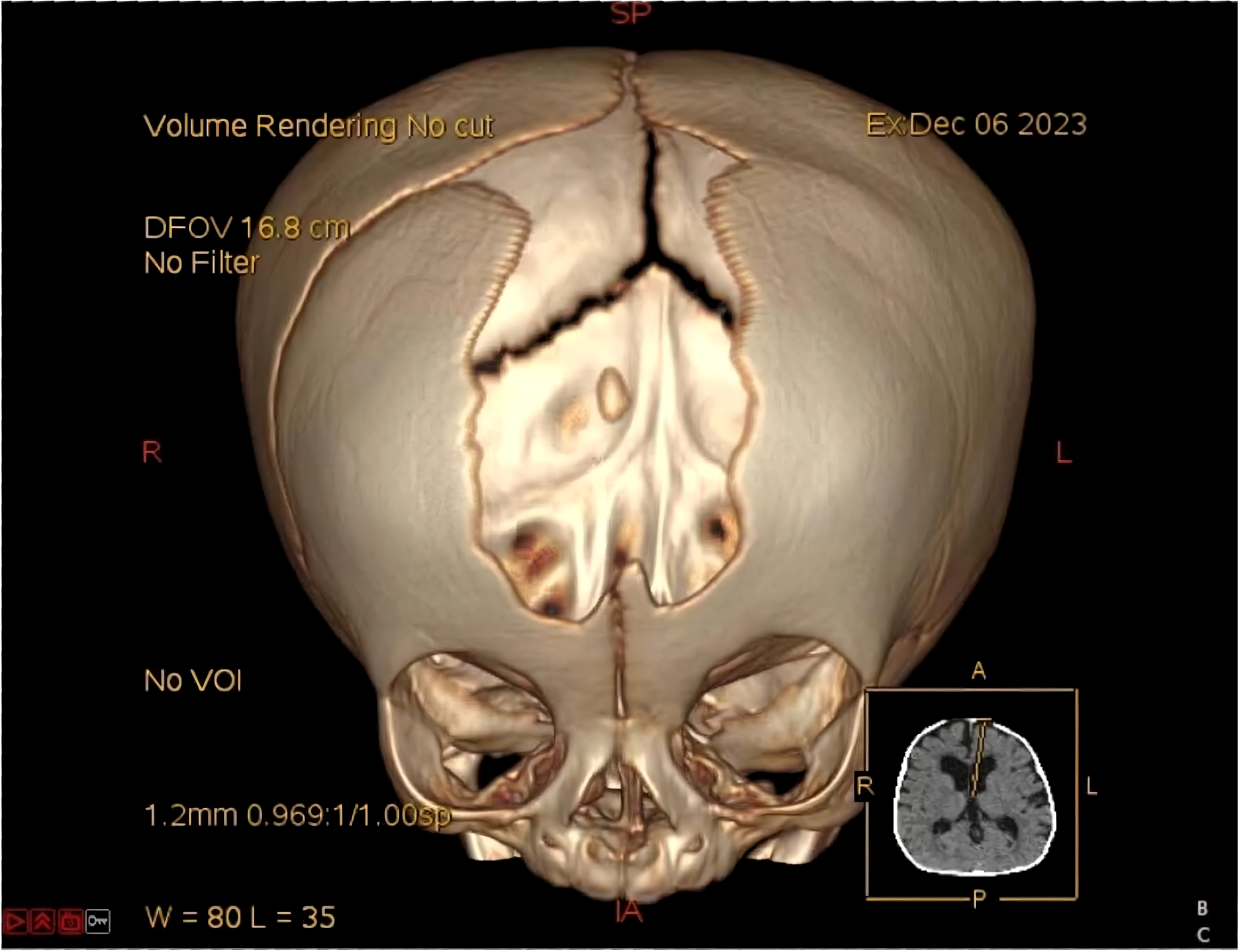
Three-dimensional cranial CT imaging of the patient.

Cranial computed tomography (CT) revealed abnormal cranial morphology characterized by brachycephaly, with a cephalic index (CI) of approximately 100%, and significant craniofacial disproportion. Partial defects of the bilateral frontal bones were observed. Premature fusion was confirmed in the left coronal suture, while the lambdoid, squamosal, and temporoparietal sutures were patent. Radiographs of the elbows demonstrated complete osseous fusion between the distal humerus and the proximal radii, with aplasia of the radial head ossification centers. Malalignment between the radius and ulna was evident bilaterally (**Figures 4**, **5**).

### Treatment and follow up

After admission, the patient was diagnosed with craniosynostosis and moderate-to-severe hydrocephalus. After completing preoperative evaluations and preparation, cranial vault reconstruction surgery was performed without complication. Postoperative recovery was favorable, with intracranial pressure approaching normal levels, and the patient was discharged in stable condition. However, the patient was lost to follow-up, and the current clinical status remains unknown.

### Pathogenicity of two POR variants

**Figure 6:**
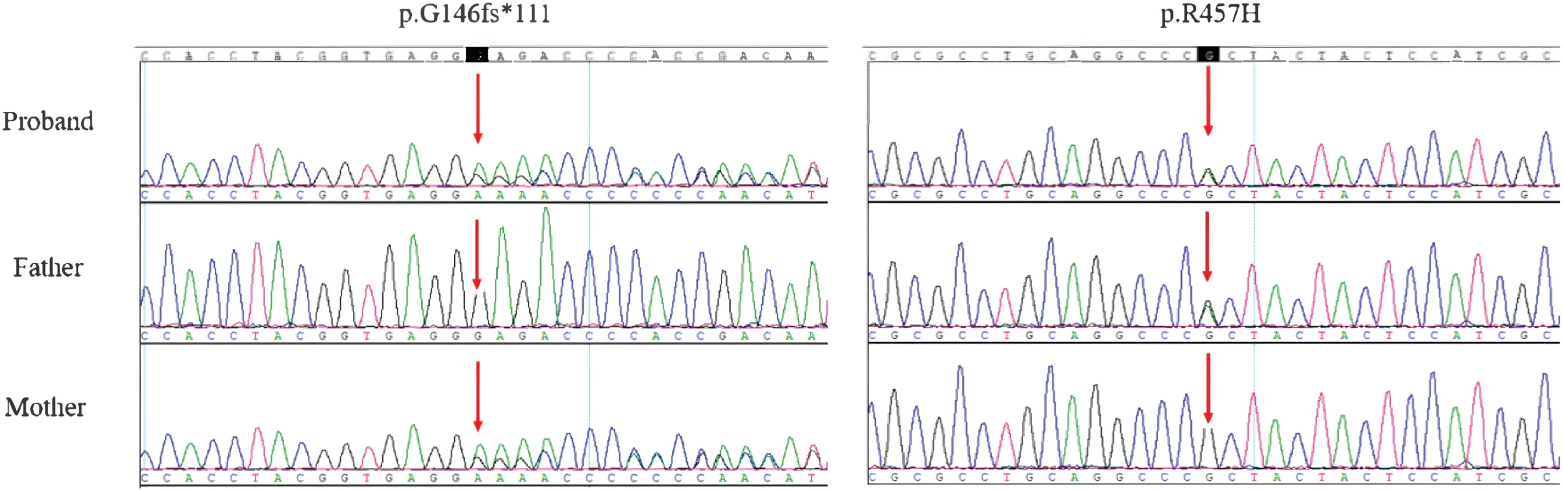
Sanger sequencing analysis of POR in the proband and parents. (The arrows indicated the mutated nucleotides. The patient carries a compound heterozygous frameshift variant (p.G146fs*111) and a missense variant (p.R457H) in POR. p.R457H was inherited from proband’s heterozygous father and p.G146fs*111 was inherited from proband’s heterozygous mother).

Karyotype analysis revealed a normal female chromosomal pattern (46, XX). Whole-exome sequencing achieved 100.00% mean coverage with an average depth exceeding 277.55×. We identified two compound heterozygous variants in the *POR* gene of the proband: a maternally inherited frameshift variant (p.G146fs*111) and a paternally inherited missense variant (p.R457H) (**Table 1**). Both variants were validated by Sanger sequencing in the proband and his parents (**Figure 6**). In silico predictive analyses were applied to assess the potential pathogenicity of these variants (**Table 2**).

**Table 1:** Two genetic variants of POR in the proband.

Gene	Transcript	cDNA	Protein	Heterozygosity	Co-segregation	1000 genomes project	ESP6500	ExAC	GnomAD	GnomAD-EAS
POR	NM_000941.2	c.437delG	p.G146fs*111	Heterozygous	Proband andher mother	–	–	–	–	–
POR	NM_000941.2	c.1370G>A	p.R457H	Heterozygous	Proband and her father	0.0002	–	0.000076	0.000052	0.000563

“ - “: not recorded in the database.

**Table 2:** Predicted pathogenicity of the two genetic variants of POR.

Gene	cDNA, Protein	SIFT	MutationTaster	Condel	SpliceAI	dbscSNV_RF	dbscSNV_ADA	PhyloP Vertebrates	PhyloP Placetal Mammals	GERP++
POR	c.437delG, p.G146fs*111	–	–	–	polymorphic	–	–	Not conserved	Not conserved	–
POR	c.1370G>A, p.R457H	Deleterious	Disease causing	deleterious	polymorphic	–	–	conserved	Not conserved	conserved

“-”, not available or not computed.

The p.G146fs*111 variant represents a novel frameshift mutation resulting from a deletion of a guanine at nucleotide position 437. This alteration introduces a frameshift at glycine 146, generating a premature termination codon 111 amino acids downstream in the altered reading frame. According to the ACMG/AMP guidelines, this variant was classified as pathogenic (PVS1 + PM2 + PM3_Supporting) (8).

The p.R457H is a previously documented missense mutation caused by a guanine to adenine substitution at nucleotide 1370, resulting in an arginine to histidine substitution at codon 457. This variant is extremely rare, with an allele frequency of 0.0002 in the 1000 Genomes Project. Based on the ACMG/AMP guidelines, it was classified as pathogenic (PS3 + PM2 + PM3_Strong) (8).

### Systematic review results

After a systematic literature review, 50 studies meeting the inclusion criteria were identified, encompassing a total of 167 patients with PORD. The cohort included 77 cases from East Asia (22 of which were from China) and 90 from other regions. There were 77 male and 90 female patients. The majority (124 cases, 74.25%) were infants or children. The most frequently reported clinical manifestations were hormonal abnormalities or delayed puberty (127 cases, 76.05%), skeletal deformities (124 cases, 74.25%), gonadal deformities (121 cases, 72.46%), and adrenal insufficiency or crisis (108 cases, 64.67%). Among female patients, ovarian cysts were documented in 36 cases (40.00%). At the genetic level, the p.R457H and p.A287P variants were the most prevalent. The allele frequency of p.A287P was 14.97%, while that of p.R457H reached 38.64%. This latter variant was also the most common in the Chinese subgroup (allele frequency: 38.64%) (**Table 3**).

**Table 3:** Summary of reported PORD cases worldwide.

No.of patients	Sex (male/female)	Age at diagnosis (Infant/Child/Adult)	Region (East Asia/Others)	Skeletal deformities	Gonadal deformities	Hormonal abnormalities or delayed puberty	Adrenal insufficiency or crisis	Ovarian cysts	POR genotype	References
4/4	2/2	0/3/1	0/4	3	3	3	4	1	p.A287P (2/8); p.R457H (1/8); c.731 + 1G>A (1/8); p.V492E (1/8); p.C569Y (1/8); p.V608F (1/8)	(9)
2/2	1/1	0/2/0	2/0	–	1	1	2	0	p.R457H (2/4); c.1329insC (1/4); c.1698insC (1/4)	(10)
1/1	1/0	0/0/1	0/1	1	0	0	1	0	p.A284P (2/2)	(11)
19/38	11/8	0/19/0	3/16	19	12	10	5	0	p.A287P (10/34); p.R457H (7/34); p.G539R (1/34); p.F646del (1/34); p.M263V (1/34); p.Q153R (1/34); p.Q153R (1/34); p.Y459H (1/34); p.Y459H (1/34); p.R616X (1/34); p.L565P (1/34); p.T142A (1/34); p.A115V (1/34); c.580_581insTACGTGGACAAGC (1/34); c.1551_1552insTGCCCATGTTCGTGCGC (1/34); c. 1621_1622insC (1/34); c. 1348_1349ins GAGC (1/34); c. IVS6_2ArT(1/34); c. IVS7(2_3)insT (1/34); c.1619_1620insCCTTCAAGGCCACCACGCCTGTCATCATGATGGGCCCCGGCACCGGGGT (1/34)	(12)
2/9	1/1	2/0/0	0/2	–	1	0	2	0	p.Y178D (2/4); p.C566Y (2/4)	(13)
4/4	4/0	1/2/1	0/4	0	4	4	4	0	p.G539R (8/8)	(14)
35/35	16/19	0/21/14	35/0	28	26	35	10	8	p.R457H (42/70)	(15)
1/3	0/1	0/1/0	1/0	1	1	1	1	0	p.T228I	(16)
4/4	1/3	0/2/2	0/4	3	3	4	4	0	p.A287P (1/8); c.delGGA651-653(delE217) (1/8); p.N185K (1/8); p.L577R (1/8); p.G539R (2/8); c.1363delC (1/8); c.697-698insGAAC (1/8)	(17)
1/1	0/1	0/1/0	1/0	1	1	1	1	0	p.R457H (2/2)	(18)
7/7	2/5	0/3/4	1/6	7	4	4	6	5	p.A287P (6/14); p.Y376LfsZ74 (1/14); p.T142A (1/14); p.R223X (1/14); p.R457H (1/14); p.Y576X (1/14); c.IVS7_dupT (1/14); c.32062delG (1/14); c.32171A>G (1/14)	(19)
2/2	0/2	2/0/0	0/2	2	2	2	2	0	p.P399_E401del (4/4)	(20)
28/30	12/16	21/4/3	0/28	25	20	26	24	4	p.R457H (2/56); p.A287P (26/56); p.C569Y (2/56); p.Y181D (2/56); c. IVS6_2A>T (2/56); p.V472AfsX102 (1/56); p.Q455RfsX544 (1/56); p.IVS7 + 2dupT (1/56); p. H628P (1/56); c.Del ex U1–1 (1/56); p.IVS8 + 1G>A (1/56); p.I444HfsX6 (1/56); p. Y87X (1/56); p. Y576X (1/56); p.Y607C (1/56); p.E601SfsX12 (1/56); p. R498P (2/56); p.Y376LfsX74 (1/56); p.T142A (1/56); p. R616X (1/56); p.IVS7 + 2dupT (1/56); p.R223X(1/56); c.Dup ex 2_5(1/56)	(21)
1/1	0/1	0/1/0	0/1	0	1	0	1	0	p.G539R (1/2); p.G80R (1/2)	(22)
1/1	1/0	0/1/0	0/1	1	1	1	0	0	c.859G>C (2/2)	(23)
1/1	0/1	TOP	0/1	1	1	–	–	–	p.A287P (1/2); c.732A>T (1/2)	(24)
1/3	0/1	TOP	0/1	1	1	–	–	–	c.859G>C (2/2)	(25)
1/1	1/0	0/0/1	0/1	0	1	1	–	–	p.del531Val (1/5); p.G858C (1/5); p.A259G (1/5); p.A503V (1/5); p.S572S (1/5)	(26)
1/1	0/1	1/0/0	0/1	0	1	1	1	0	p.L374H (1/2); c.5 + 4A>G (1/2)	(27)
1/1	0/1	0/1/0	0/1	1	0	1	1	1	p.A287P (2/2)	(28)
1/1	1/0	0/0/1	1/0	1	1	1	1	–	p.G88S (1/2); p.R457H (1/2)	(29)
1/1	0/1	0/0/1	1/0	0	1	1	1	1	p.R457H (2/2)	(4)
1/1	0/1	0/1/0	0/1	1	1	1	1	1	p.G144S (1/2); p.W422X (1/2)	(30)
1/1	0/1	0/0/1	1/0	0	1	1	1	1	p.Y326D (2/2)	(31)
2/2	0/2	0/1/1	2/0	0	1	1	2	0	p.R457H (2/4); p.R223X (1/4); p.Y607C (1/4)	(32)
1/1	0/1	0/1/0	1/0	1	1	1	1	0	c.744C>G (1/2); c.1370G>A (1/2)	(33)
8/8	5/3	0/8/0	8/0	7	8	8	8	1	p.R457H (9/16); p.Y248X (1/16); p.Y248X (1/16); p.R554X (1/16); p.Y607C (1/16); p.D210G (1/16); c.517-19_517-10delGGCCCCTGTGinsC (1/16); c.517-19_517-10delGGCCCCTGTGinsC (1/16)	(34)
1/1	0/1	0/1/0	0/1	0	1	0	1	0	p.L25Ffs*93 (1/2); p.R550W (1/2)	(35)
5/5	0/5	0/0/5	0/5	1	1	3	5	5	p.Q609* (2/10); p.W620S (2/10); p.A287P (2/10); p.P442S (1/10); p.R550W (1/10); c.1249-IG>C(p.)? (1/10)	(36)
1/1	0/1	0/0/1	0/1	1	1	1	1	1	p.T142A (1/2); p.Y376LfsX74 (1/2)	(37)
4/4	2/2	0/4/0	4/0	2	4	2	4	0	p.R457H (6/8); p.I444Hfs*6 (2/8);	(5)
1/1	0/1	0/0/1	1/0	0	1	1	0	1	p.399_401delPSE (1/2); c.IVS14-1G>G/C (1/2)	(38)
1/1	1/0	0/1/0	1/0	1	1	1	1	–	p.R457H (1/2); c.517-19_517-10delGGCCCCTGTGinsC (1/2)	(39)
1/1	0/1	0/1/0	1/0	1	1	1	1	1	p.R457H (2/2)	(40)
1/1	1/0	0/1/0	0/1	0	0	1	1	–	–	(41)
2/2	1/1	0/2/0	0/2	2	1	1	2	0	p.I310_S313delinsT (2/2)	(42)
1/1	0/1	0/1/0	1/0	0	0	1	1	1	p.R457H (1/2); p.P399_E401del (1/2)	(43)
1/8	0/1	0/1/0	0/1	0	1	1	0	0	p.G539R (2/2)	(44)
1/1	0/1	0/1/0	1/0	1	0	1	1	1	c.1370G>A (1/2)	(45)
2/2	1/1	0/2/0	2/0	2	2	–	–	0	p.R457H (2/4); c.760 + 1 G>A (1/4); c.396–1 G>A (1/4)	(46)
1/5	1/0	0/1/0	1/0	0	1	0	1	0	p. A541V (1/2); p. Q602 *(1/2)	(47)
1/1	0/1	1/0/0	1/0	1	1	0	0	0	p.R457H (1/2); c.426 + 1_538–1del (1/2)	(48)
1/1	1/0	0/1/0	0/1	0	0	0	1	0	p.Val631Ile (1/2); c.516G>A (1/2)	(49)
1/1	1/0	1/0/0	1/0	1	0	1	1	–	p.R457H (2/2)	(50)
2/23	1/1	1/0/1	2/0	1	2	0	0	1	p.G88S (2/4); p. R457H (1/4); p. G537S (1/4)	(2)
2/2	0/2	0/0/2	2/0	1	1	2	0	2	p.R457H (1/4); p.L454P (1/4); c.1631T>C (1/4); c.1723G>A (1/4)	(51)
1/1	0/1	0/1/0	1/0	1	–	–	–	–	p.R554X (1/2); p.R457H (1/2)	(52)
1/22	0/1	1/0/0	1/0	1	1	0	1	0	p.R457H (2/2)	(53)
1/1	0/1	0/1/0	0/1	1	1	1	1	0	p.P452L(2/2)	(54)
2/3	0/2	1/0/0, TOP	0/2	2	2	–	1	–	p.E217del (4/4)	(55)

“TOP”: termination of pregnancy; “ - “: not mentioned.

## Discussion

In 1985, Peterson RE et al. described a case of congenital adrenal hyperplasia (CAH) exhibiting combined features of 21-hydroxylase deficiency (21-OHD) and 17α-hydroxylase deficiency (17-OHD), yet no pathogenic variants were identified in CYP21A2 or CYP17A1 (3, 56). It was not until 2004 that Flück CE et al. demonstrated that this novel form of CAH was caused by mutations in the POR gene, which led to impaired activities of multiple CYP enzymes, including CYP21A2, CYP17A1, and CYP19A1 (9). As an essential electron transfer protein, POR supports the function of numerous CYP enzymes involved in steroidogenesis, cholesterol metabolism, and xenobiotic detoxification (**Figure 1**). The extent of enzymatic deficiency and corresponding clinical manifestations of PORD vary considerably depending on the specific POR mutation, contributing to significant phenotypic heterogeneity (1). Current literature on PORD remains limited, predominantly consisting of isolated case reports or small retrospective series, many of which are outdated and offer limited insight into modern diagnostic or therapeutic approaches. In the present study, we first delineated the clinical and paraclinical profile of a proband harboring a novel POR variant (p.G146fs*111), identified via WES. Furthermore, by systematically reviewing eligible publications, we assembled a cohort of 167 PORD patients with a male-to-female ratio of 77:90. The majority of cases were diagnosed during infancy or childhood. The most common clinical features included hormonal abnormalities or delayed puberty (76.05%), skeletal deformities (74.25%), gonadal deformities (72.46%), and adrenal insufficiency or crisis (64.67%). Ovarian cysts were present in 40.00% of female patients. Genetically, the p.R457H and p.A287P variants were the most frequent. Notably, the p.R457H variant exhibited an allele frequency of 38.64% in Chinese patients, markedly higher than that observed in other populations.

Most patients with PORD exhibit skeletal deformities resembling Antley-Bixler syndrome, such as craniosynostosis, midface hypoplasia, arachnodactyly, elbow synostosis, and femoral bowing (1, 3, 42, 57). The proband in this study presented with premature fusion of the left coronal suture, midface hypoplasia, and humeroradial synostosis. Our systematic review found that skeletal malformations occurred in 74.25% of patients, aligning with previous reports. The severity of these malformations correlates with the underlying POR mutation. Krone N et al. developed a scoring system to quantify the severity of skeletal anomalies based on their type and location, demonstrating that patients with mild to moderate deformities were typically compound heterozygotes for missense variants, whereas those with severe malformations often carried loss-of-function mutations such as frameshift or splice-site variants (21). Although earlier studies suggested that the p.R457H variant is associated with more severe skeletal involvement, a study by Dean B et al. reported that patients homozygous for p.R457H did not display more pronounced skeletal defects than those carrying p.R457H in combination with other variants or compared to carriers of p.A287P (17, 34). Other missense variants, including p.C569Y, p.G539R, p.L577R, and p.Y326D, also generally resulted in milder skeletal phenotypes (1). These conflicting findings underscore persistent controversy in genotype–phenotype correlations, highlighting the need for larger-scale studies. The mechanism underlying skeletal malformations in PORD remains incompletely elucidated. One proposed pathway involves impaired cholesterol synthesis in chondrocytes due to deficient activity of lanosterol 14-α-demethylase (CYP51A1) and squalene monooxygenase (SQLE), leading to aberrant differentiation and increased apoptosis, which ultimately disrupts skeletal development (42, 58). Although the skeletal features of PORD closely mimic those of Antley-Bixler syndrome (ABS), accurate discrimination between these disorders is critical due to distinct management strategies. Elevated steroid hormone levels and the presence of genital anomalies in PORD provide key diagnostic clues for differentiation (7).

**Figure 7:**
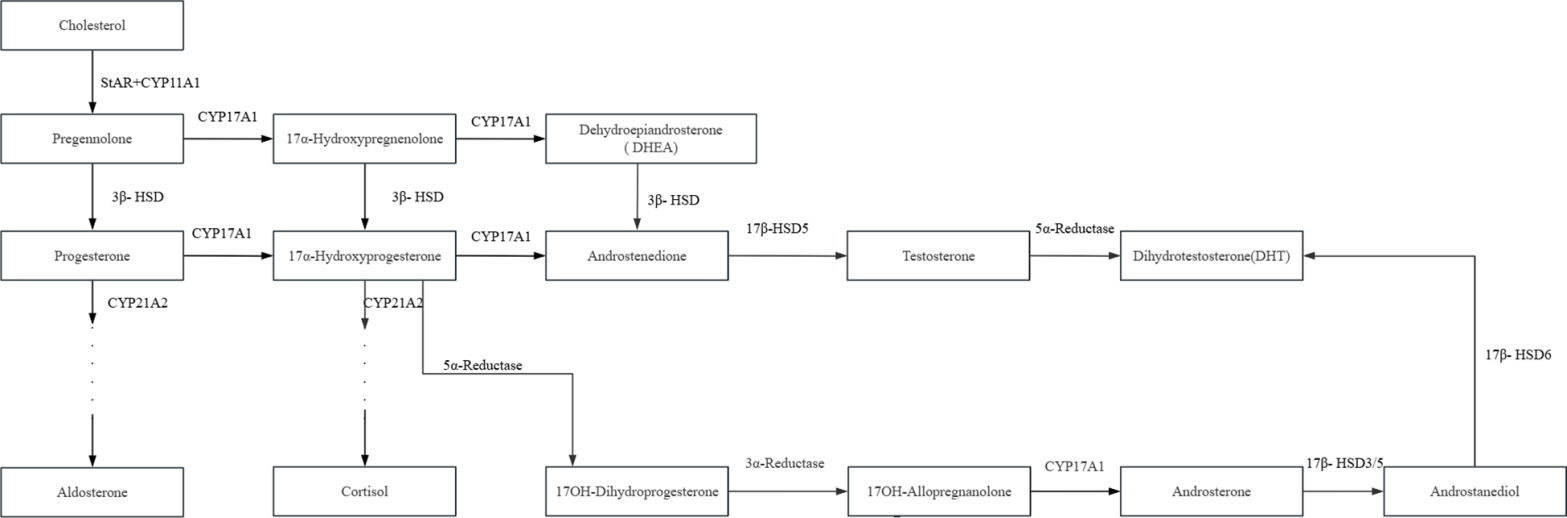
Schematic diagram of androgen synthesis via the “Backdoor Pathway” in female fetuses.

In PORD, impaired function of enzymes such as CYP17A1, CYP19A1, and CYP21A2 disrupts the synthesis of testosterone and dihydrotestosterone, as well as the conversion of androgens to estrogens, resulting in a spectrum of genital anomalies (4, 32, 34, 59). In male patients, deficient fetal testosterone production leads to undervirilization at birth, commonly presenting as micropenis or hypospadias, and occasionally cryptorchidism (4). In female patients, fetal CYP17A1 deficiency promotes accumulation of adrenal 17α-hydroxyprogesterone, which is then shunted into the “backdoor pathway” to produce elevated levels of the potent androgen dihydrotestosterone (**Figure 7**) (34). This mechanism induces virilization of external genitalia—such as clitoromegaly and labial fusion—at birth, and may also cause maternal virilization during pregnancy, including hirsutism and acne (60). Multiple studies consistently report that abnormal sexual development is generally more pronounced in female than in male patients with PORD (21, 42). For example, in a clinical series by Fukami M et al. including 35 patients, all genotypic females exhibited genital anomalies, compared to only 43.7% of genotypic males who presented with abnormalities such as micropenis or hypospadias (15). Furthermore, among known POR variants, p.R457H is most frequently associated with maternal virilization during gestation (1, 61). After birth, shifts in adrenal steroidogenic enzyme expression and a decline in 17α-hydroxyprogesterone close the backdoor pathway, leading to persistently low androgen levels and arrest of further virilization (32, 62). In the present case, the proband exhibited clitoromegaly at birth, and her mother developed hirsutism and acne during pregnancy, which aligns closely with this recognized clinical trajectory. As patients progress into puberty and adulthood, disrupted biosynthesis of sex steroids commonly manifests as delayed puberty, incomplete or absent development of secondary sexual characteristics, and infertility in both sexes. Females often present with menstrual disturbances—including oligomenorrhea or amenorrhea—and are frequently affected by ovarian cysts. The majority of affected female patients develop ovarian cysts, a phenomenon attributed to several endocrine and enzymatic disturbances. Specifically, estrogen deficiency leads to hypergonadotropic hypogonadism, wherein elevated gonadotropins persistently stimulate the ovaries, contributing to cystic formation. Additionally, deficient CYP51A1 activity may impair the production of meiosis-activating sterols, which are essential for normal oocyte maturation and follicular development. These pathophysiological mechanisms collectively explain the high prevalence and clinical severity of ovarian cysts in PORD, which often necessitate a combination of surgical intervention and hormonal replacement therapy (19).

Baseline adrenocorticotropic hormone (ACTH) and cortisol levels in patients with PORD are frequently observed to be within normal limits. However, glucocorticoid biosynthesis in the adrenal glands is impaired due to deficiencies in CYP21A2 and CYP17A1 activity, patients to adrenal crisis under stressful conditions such as infection, trauma, or surgery (42, 57). In the study by Bai Y et al., approximately 74.6% of PORD patients showed an insufficient cortisol response during the ACTH stimulation test, indicating compromised adrenal reserve (4). This underscores the critical diagnostic utility of ACTH stimulation test in identifying subclinical adrenal insufficiency in this population. Glucocorticoid replacement therapy should be individualized based on clinical manifestations and biochemical findings. While approximately half of all PORD patients require lifelong hydrocortisone replacement, others may only need supplemental doses during periods of physiologic stress (21, 28, 34). Although mineralocorticoid excess and consequent hypertension are uncommon in the overall PORD population, several studies have reported a higher incidence of hypertension among carriers of the p.A287P variant (63, 64). Thus, regular blood pressure monitoring is recommended, particularly in this genetic subgroup. Furthermore, the reduced activity of hepatic CYP enzymes—including CYP1A2, CYP2C19, and CYP3A4—can significantly alter drug metabolism in PORD patients (7). Of particular clinical relevance, CYP3A4 is responsible for metabolizing nearly 50% of commonly prescribed medications (65, 66). Consequently, clinicians should carefully evaluate potential pharmacokinetic alterations and consider dose adjustments when administering drugs that are substrates of these enzymes.

To date, more than 200 distinct mutations and single-nucleotide polymorphisms (SNPs) in the POR gene have been documented, encompassing missense, nonsense, insertion, deletion, duplication, and splice-site mutations, in addition to submicroscopic deletions affecting one or more exons (67). In the present case, the patient carried compound heterozygous mutations, p.G146fs*111 and p.R457H. The p.G146fs*111 mutation has not been previously documented, representing a novel variant that expands the mutational spectrum of POR. Previous studies have consistently identified p.A287P as one of the most prevalent POR variants in Caucasian populations. In contrast, large-scale analyses of hotspot POR mutations in East Asian populations remain limited, and definitive conclusions have yet to be established. Japan has conducted relatively early research on PORD; a 2009 study by Fukami et al. identified p.R457H as the most frequent variant, with an allele frequency of 67% (15). This high prevalence was later corroborated in a nationwide Japanese study by Yatsuga et al. (68). In Korea, all six reported PORD cases carried the p.R457H variant (5). Among the 22 documented Chinese PORD cases included in our analysis, the allele frequency of the p.R457H variant was 38.64%. Together, these findings strongly suggest that p.R457H is a common pathogenic variant in East Asian populations. Different POR mutations differentially impair the activities of distinct P450 enzymes, thereby contributing to heterogeneous clinical phenotypes. The p.R457H variant leads to a near-complete loss of CYP19A1 and CYP17A1 activities, whereas p.A287P reduces 17α-hydroxylase and 17,20-lyase activities to approximately 20% and 10% of wild-type levels, respectively, while largely sparing CYP19A1 function (34). Accordingly, patients carrying the p.R457H variant tend to exhibit more severe skeletal manifestations and a higher incidence of maternal virilization during pregnancy than those with the p.A287P variant. However, given the considerable complexity of the POR mutational spectrum, further functional and clinical studies are essential to fully elucidate genotype–phenotype correlations.

This study has two main limitations. First, the case information was obtained through a retrospective review of patient medical records, which inherently depends on the completeness and accuracy of these documents. Second, our analysis incorporated data extracted from previously published studies rather than direct patient record review, which similarly relies on the accuracy and thoroughness of reporting in the existing literature. Furthermore, the patient cohort in our study may not be fully representative of the entire spectrum of PORD, as individuals with more severe manifestations are more likely to seek medical care and be reported, while those with milder symptoms are often overlooked or remain undiagnosed. This potential bias may be further exacerbated in underserved regions with limited healthcare access and diagnostic capabilities. Therefore, larger-scale and scientifically rigorous prospective clinical studies are needed in the future to validate and extend our findings.

In conclusion, PORD is a rare autosomal recessive disorder that is frequently misdiagnosed. We have described the clinical presentation of a PORD patient in whom WES revealed a novel mutation (p.G146fs*111), thereby expanding the known mutational spectrum of the POR gene. Through a systematic review of the literature, we identified and analyzed a total of 167 reported PORD cases, summarizing the most common clinical manifestations to facilitate early diagnosis. Our analysis further indicates that p.R457H may represent a potential hotspot mutation in East Asian populations. A multidisciplinary management approach is recommended, including individualized glucocorticoid replacement, regular blood pressure monitoring, and surgical correction of skeletal or genital abnormalities when indicated. Furthermore, the close relationship between genotype and phenotype in PORD highlights the utility of WES for genetic diagnosis, thereby improving diagnostic accuracy, guiding clinical management, and enabling informed genetic counseling for affected families.

## Data Availability

The datasets presented in this study can be found in online repositories. The names of the repository/repositories and accession number(s) can be found in the article/**Supplementary Material**.
