# Supplementary material for: Case Report: Integrating clinical presentation and genetic analysis in P450 oxidoreductase deficiency: a novel mutation and systematic review
Source: Front Endocrinol (Lausanne). 2026 Apr 10;17:1791297. doi: 10.3389/fendo.2026.1791297 (PMC13105989; doi:10.3389/fendo.2026.1791297)
Supplement: Supplementary file 1 [file Table1.docx]

**TABLE S1** Summary of reported PORD cases worldwide

| No.of  Patients | Sex  (male/female) | Age at Diagnosis (Infant/Child/Adult) | Region (East Asia/Others) | Skeletal  deformities | DSD | Hormonal abnormalities or  delayed puberty | Adrenal  insufficiency or  crisis | Ovarian  cysts | POR genotype | References |
| --- | --- | --- | --- | --- | --- | --- | --- | --- | --- | --- |
| 4/4 | 2/2 | 0/3/1 | 0/4 | 3 | 3 | 3 | 4 | 1 | p.A287P (2/8); p.R457H (1/8); c.731+1G>A (1/8); p.V492E (1/8);  p.C569Y (1/8); p.V608F (1/8) | (9) |
| 2/2 | 1/1 | 0/2/0 | 2/0 | - | 1 | 1 | 2 | 0 | p.R457H (2/4); c.1329insC (1/4); c.1698insC (1/4) | (10) |
| 1/1 | 1/0 | 0/0/1 | 0/1 | 1 | 0 | 0 | 1 | 0 | p.A284P (2/2) | (11) |
| 19/38 | 11/8 | 0/19/0 | 3/16 | 19 | 12 | 10 | 5 | 0 | p.A287P (10/34); p.R457H (7/34); p.G539R (1/34); p.F646del (1/34); p.M263V (1/34); p.Q153R (1/34); p.Q153R (1/34); p.Y459H (1/34); p.Y459H (1/34); p.R616X (1/34); p.L565P (1/34); p.T142A (1/34); p.A115V (1/34); c.580_581insTACGTGGACAAGC (1/34); c.1551_1552insTGCCCATGTTCGTGCGC (1/34); c. 1621_1622insC (1/34);  c. 1348_1349ins GAGC (1/34); c. IVS6_2ArT(1/34); c. IVS7(2_3)insT (1/34); c.1619_1620insCCTTCAAGGCCACCACGCCTGTCATCATGATGGGCCCCGGCACCGGGGT (1/34) | (12) |
| 2/9 | 1/1 | 2/0/0 | 0/2 | - | 1 | 0 | 2 | 0 | p.Y178D (2/4); p.C566Y (2/4) | (13) |
| 4/4 | 4/0 | 1/2/1 | 0/4 | 0 | 4 | 4 | 4 | 0 | p.G539R (8/8) | (14) |
| 35/35 | 16/19 | 0/21/14 | 35/0 | 28 | 26 | 35 | 10 | 8 | p.R457H (42/70) | (15) |
| 1/3 | 0/1 | 0/1/0 | 1/0 | 1 | 1 | 1 | 1 | 0 | p.T228I | (16) |
| 4/4 | 1/3 | 0/2/2 | 0/4 | 3 | 3 | 4 | 4 | 0 | p.A287P (1/8); c.delGGA651-653(delE217) (1/8); p.N185K (1/8); p.L577R (1/8); p.G539R (2/8); c.1363delC (1/8); c.697-698insGAAC (1/8) | (17) |
| 1/1 | 0/1 | 0/1/0 | 1/0 | 1 | 1 | 1 | 1 | 0 | p.R457H (2/2) | (18) |
| 7/7 | 2/5 | 0/3/4 | 1/6 | 7 | 4 | 4 | 6 | 5 | p.A287P (6/14); p.Y376LfsZ74 (1/14); p.T142A (1/14); p.R223X (1/14); p.R457H (1/14); p.Y576X (1/14); c.IVS7_dupT (1/14); c.32062delG (1/14); c.32171A>G (1/14) | (19) |
| 2/2 | 0/2 | 2/0/0 | 0/2 | 2 | 2 | 2 | 2 | 0 | p.P399_E401del (4/4) | (20) |
| 28/30 | 12/16 | 21/4/3 | 0/28 | 25 | 20 | 26 | 24 | 4 | p.R457H (2/56); p.A287P (26/56); p.C569Y (2/56); p.Y181D (2/56); c. IVS6_2A>T (2/56); p.V472AfsX102 (1/56); p.Q455RfsX544 (1/56); p.IVS7+2dupT (1/56); p. H628P (1/56); c.Del ex U1–1 (1/56); p.IVS8+1G>A (1/56); p.I444HfsX6 (1/56); p. Y87X (1/56); p. Y576X (1/56); p.Y607C (1/56); p.E601SfsX12 (1/56); p. R498P (2/56); p.Y376LfsX74 (1/56); p.T142A (1/56); p. R616X (1/56); p.IVS7+2dupT (1/56); p.R223X(1/56); c.Dup ex 2_5(1/56) | (21) |
| 1/1 | 0/1 | 0/1/0 | 0/1 | 0 | 1 | 0 | 1 | 0 | p.G539R (1/2); p.G80R (1/2) | (22) |
| 1/1 | 1/0 | 0/1/0 | 0/1 | 1 | 1 | 1 | 0 | 0 | c.859G>C (2/2) | (23) |
| 1/1 | 0/1 | TOP | 0/1 | 1 | 1 | - | - | - | p.A287P (1/2); c.732A>T (1/2) | (24) |
| 1/3 | 0/1 | TOP | 0/1 | 1 | 1 | - | - | - | c.859G>C (2/2) | (25) |
| 1/1 | 1/0 | 0/0/1 | 0/1 | 0 | 1 | 1 | - | - | p.del531Val (1/5); p.G858C (1/5); p.A259G (1/5); p.A503V (1/5); p.S572S (1/5) | (26) |
| 1/1 | 0/1 | 1/0/0 | 0/1 | 0 | 1 | 1 | 1 | 0 | p.L374H (1/2); c.5+4A>G (1/2) | (27) |
| 1/1 | 0/1 | 0/1/0 | 0/1 | 1 | 0 | 1 | 1 | 1 | p.A287P (2/2) | (28) |
| 1/1 | 1/0 | 0/0/1 | 1/0 | 1 | 1 | 1 | 1 | - | p.G88S (1/2); p.R457H (1/2) | (29) |
| 1/1 | 0/1 | 0/0/1 | 1/0 | 0 | 1 | 1 | 1 | 1 | p.R457H (2/2) | (4) |
| 1/1 | 0/1 | 0/1/0 | 0/1 | 1 | 1 | 1 | 1 | 1 | p.G144S (1/2); p.W422X (1/2) | (30) |
| 1/1 | 0/1 | 0/0/1 | 1/0 | 0 | 1 | 1 | 1 | 1 | p.Y326D (2/2) | (31) |
| 2/2 | 0/2 | 0/1/1 | 2/0 | 0 | 1 | 1 | 2 | 0 | p.R457H (2/4); p.R223X (1/4); p.Y607C (1/4) | (32) |
| 1/1 | 0/1 | 0/1/0 | 1/0 | 1 | 1 | 1 | 1 | 0 | c.744C>G (1/2); c.1370G>A (1/2) | (33) |
| 8/8 | 5/3 | 0/8/0 | 8/0 | 7 | 8 | 8 | 8 | 1 | p.R457H (9/16); p.Y248X (1/16); p.Y248X (1/16); p.R554X (1/16); p.Y607C (1/16); p.D210G (1/16); c.517-19_517-10delGGCCCCTGTGinsC (1/16); c.517-19_517-10delGGCCCCTGTGinsC (1/16) | (34) |
| 1/1 | 0/1 | 0/1/0 | 0/1 | 0 | 1 | 0 | 1 | 0 | p.L25Ffs*93 (1/2); p.R550W (1/2) | (35) |
| 5/5 | 0/5 | 0/0/5 | 0/5 | 1 | 1 | 3 | 5 | 5 | p.Q609* (2/10); p.W620S (2/10); p.A287P (2/10); p.P442S (1/10); p.R550W (1/10); c.1249-IG>C(p.?) (1/10) | (36) |
| 1/1 | 0/1 | 0/0/1 | 0/1 | 1 | 1 | 1 | 1 | 1 | p.T142A (1/2); p.Y376LfsX74 (1/2) | (37) |
| 4/4 | 2/2 | 0/4/0 | 4/0 | 2 | 4 | 2 | 4 | 0 | p.R457H (6/8); p.I444Hfs*6 (2/8); | (5) |
| 1/1 | 0/1 | 0/0/1 | 1/0 | 0 | 1 | 1 | 0 | 1 | p.399_401delPSE (1/2); c.IVS14-1G>G/C (1/2) | (38) |
| 1/1 | 1/0 | 0/1/0 | 1/0 | 1 | 1 | 1 | 1 | - | p.R457H (1/2); c.517-19_517-10delGGCCCCTGTGinsC (1/2) | (39) |
| 1/1 | 0/1 | 0/1/0 | 1/0 | 1 | 1 | 1 | 1 | 1 | p.R457H (2/2) | (40) |
| 1/1 | 1/0 | 0/1/0 | 0/1 | 0 | 0 | 1 | 1 | - | - | (41) |
| 2/2 | 1/1 | 0/2/0 | 0/2 | 2 | 1 | 1 | 2 | 0 | p.I310_S313delinsT (2/2) | (42) |
| 1/1 | 0/1 | 0/1/0 | 1/0 | 0 | 0 | 1 | 1 | 1 | p.R457H (1/2); p.P399_E401del (1/2) | (43) |
| 1/8 | 0/1 | 0/1/0 | 0/1 | 0 | 1 | 1 | 0 | 0 | p.G539R (2/2) | (44) |
| 1/1 | 0/1 | 0/1/0 | 1/0 | 1 | 0 | 1 | 1 | 1 | c.1370G>A (1/2) | (45) |
| 2/2 | 1/1 | 0/2/0 | 2/0 | 2 | 2 | - | - | 0 | p.R457H (2/4); c.760 + 1 G>A (1/4); c.396-1 G>A (1/4) | (46) |
| 1/5 | 1/0 | 0/1/0 | 1/0 | 0 | 1 | 0 | 1 | 0 | p. A541V (1/2); p. Q602 *(1/2) | (47) |
| 1/1 | 0/1 | 1/0/0 | 1/0 | 1 | 1 | 0 | 0 | 0 | p.R457H (1/2); c.426+1_538–1del (1/2) | (48) |
| 1/1 | 1/0 | 0/1/0 | 0/1 | 0 | 0 | 0 | 1 | 0 | p.Val631Ile (1/2); c.516G>A (1/2) | (49) |
| 1/1 | 1/0 | 1/0/0 | 1/0 | 1 | 0 | 1 | 1 | - | p.R457H (2/2) | (50) |
| 2/23 | 1/1 | 1/0/1 | 2/0 | 1 | 2 | 0 | 0 | 1 | p.G88S (2/4); p. R457H (1/4); p. G537S (1/4) | (2) |
| 2/2 | 0/2 | 0/0/2 | 2/0 | 1 | 1 | 2 | 0 | 2 | p.R457H (1/4); p.L454P (1/4); c.1631T>C (1/4); c.1723G>A (1/4) | (51) |
| 1/1 | 0/1 | 0/1/0 | 1/0 | 1 | - | - | - | - | p.R554X (1/2); p.R457H (1/2) | (52) |
| 1/22 | 0/1 | 1/0/0 | 1/0 | 1 | 1 | 0 | 1 | 0 | p.R457H (2/2) | (53) |
| 1/1 | 0/1 | 0/1/0 | 0/1 | 1 | 1 | 1 | 1 | 0 | p.P452L(2/2) | (54) |
| 2/3 | 0/2 | 1/0/0，TOP | 0/2 | 2 | 2 | - | 1 | - | p.E217del (4/4) | (55) |

“TOP”: termination of pregnancy; “ - ”: not mentioned.
